# PREVALENCE AND FACTORS ASSOCIATED WITH BACTERIAL INFECTION IN CIRRHOTIC PATIENTS IN TOGO

**DOI:** 10.1590/S0004-2803.24612024-37

**Published:** 2025-07-21

**Authors:** Laté Mawuli LAWSON-ANANISSOH, Mawunyo Henoc GBOLOU, Debehoma Venceslas REDAH, Lidawu Roland-Moise KOGOE, Yendoukoa Yves KANAKE, Aklesso BAGNY

**Affiliations:** 1 Hepato Gastroenterology Department, Campus Teaching Hospital, Lome, Togo.; 2 University of Lome, Togo.

**Keywords:** Ascites fluid, cirrhosis, infection, mortality, Togo, Ascite fluida, cirrose, infecção, mortalidade, Togo

## Abstract

**Objectives:**

to determine the prevalence and identify the factors associated with bacterial infection in cirrhotic patients in Togo.

**Methods:**

This was a descriptive and analytical cross-sectional study, with retrospective data collection, conducted in the hepato-gastroenterology department of the Campus University Hospital during three years. All patients hospitalized in the department during this period and diagnosed with cirrhosis were included in the study.

**Results:**

During the study period, 270 patients were hospitalized for cirrhosis including 63 cases of bacterial infection, a prevalence of 23.3%. Bacterial infections were represented by spontaneous infection of ascites fluid (15.9%) followed by urinary tract infection (4%). The median length of hospital stay was 10 days Thirty-six of the patients with infection died in hospital, corresponding to a mortality rate of 57.1%. Factors associated with bacterial infection were ascites (*P*=0.017; OR=4.56), hepatic encephalopathy (*P*=0.02; OR=4.32), a prothrombin level below 25% (*P*=0.002; OR=9.67) and a high MELD score (*P*=0.03; OR=0.93).

**Conclusion:**

Bacterial infection occurs in advanced cirrhosis and is associated with a poor prognosis.

## HIGHLIGHTS

• Bacterial infections are a frequent complication of cirrhosis and were associated with ascites, hepatic encephalopathy and a high MELD score.

• The most frequent type of infection in this study was spontaneous bacterial peritonitis followed by urinary tract infection.

## RESUMO

**Objetivos:**

Determinar a prevalência e identificar os fatores associados à infecção bacteriana em pacientes cirróticos no Togo.

**Métodos:**

Estudo transversal descritivo e analítico, com coleta retrospectiva de dados, realizado no serviço de hepato-gastroenterologia do Hospital Universitário Campus durante três anos. Todos os pacientes internados no serviço durante esse período e com diagnóstico de cirrose foram incluídos no estudo.

**Resultados:**

Durante o período do estudo, 270 pacientes foram hospitalizados por cirrose, incluindo 63 casos de infecção bacteriana, uma prevalência de 23,3%. As infecções bacterianas foram representadas por infecção espontânea do líquido ascite (15,9%), seguida por infecção do trato urinário (4%). A mediana do tempo de internação foi de 10 dias Trinta e seis dos pacientes com infecção morreram no hospital, correspondendo a uma taxa de mortalidade de 57,1%. Os fatores associados à infecção bacteriana foram ascite (*P*=0,017; OR=4,56), encefalopatia hepática (*P*=0,02; OR=4,32), nível de protrombina abaixo de 25% (*P*=0,002; OR=9,67) e um alto escore MELD (*P*=0,03; OR=0,93).

**Conclusão:**

A infecção bacteriana ocorre na cirrose avançada e está associada a um mau prognóstico.

## INTRODUCTION

Bacterial infections are frequent and constitute a late complication during cirrhosis. They are serious, attest to the severity of liver failure and represent a factor worsening the prognosis of cirrhosis^1^. Among hospitalized cirrhotic patients, 25 to 35% have an infection on admission or develop one during hospitalization^2^. Bacterial infections are responsible for a high mortality rate (25%) and therefore constitute a diagnostic and therapeutic emergency^1^. In Togo, there are no data on bacterial infections in cirrhotic patients. The objectives of this study were to determine the prevalence and identify the factors associated with bacterial infection in cirrhotic patients in Togo.

## METHODS

This was a descriptive and analytical cross-sectional study, with retrospective data collection, conducted in the hepato-gastroenterology department of the Campus University Hospital from January 1, 2021 to June 30, 2023. All patients hospitalized in the department during this period and diagnosed with cirrhosis on the basis of epidemiological, clinical, biological and morphological criteria were included in the study. Ascites fluid infection was defined by a neutrophil count greater than 250/mm3 in ascites fluid. Urinary tract infection was defined as the presence of a pathogenic germ in the urine. Bacterial pneumopathy was defined by the presence of an alveolar or alveolar-interstitial syndrome on imaging. Sepsis was defined by the presence of a clinical infectious syndrome and the presence of a pathogenic germ in the blood. Data were cleaned and analyzed using r software version 4.3.2 in the R studio development environment version 2023.12.1. The statistical tests used were Fischer’s exact test and chi-square test for qualitative variables; and Student’s *t-*test for quantitative variables. Logistic regression was performed to determine the factors associated with infection in cirrhotic patients. After univariate analysis, variables of clinical interest that were sufficiently associated (*P*<0.2) were introduced in a first model. Using a top-down step-by-step selection procedure with the lowest akaike’s information criterion, variables with little influence were eliminated, resulting in a reduced model. Survival curves were compared using the log rank test.

## RESULTS

**FIGURE 1:**
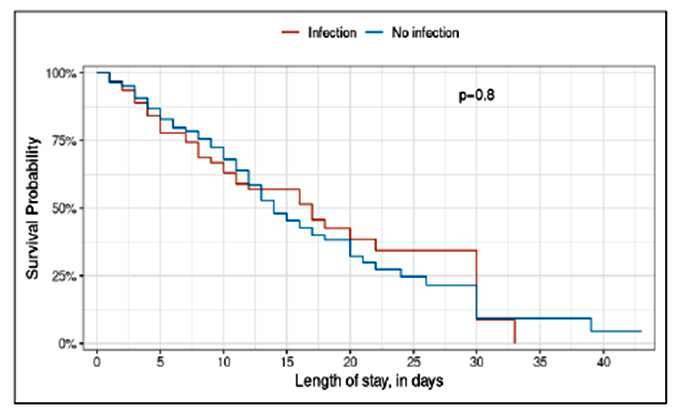
In-hospital survival of patients with and without bacterial infection.

During the study period, 270 patients were hospitalized for cirrhosis, 179 were men and 91 were women, corresponding to a sex ratio of 1.96. The reason for hospitalization was abdominal distension in 77% of cases. Clinical ascites was found in 249 patients (92.2%), and 127 patients (47%) had hepatic encephalopathy on admission. One hundred and twenty-six patients died in hospital, i.e. an in-hospital mortality rate of 46.6%. Clinical and biological characteristics and prognosis of the patients are detailed in Table 1. Sixty-three patients, i.e. 23.3% had an infectious complication during the study period. The different types of infection are detailed in Table 2. Spontaneous infection of ascites fluid was diagnosed in 43 patients i.e. 15.9%. The sex ratio was 3.3. Examination on admission revealed fever and abdominal tenderness in 25.5 and 41.8% of patients respectively. Culture of ascites fluid was performed in two cases, isolating gram-negative bacteria: Escherichia coli and Pseudomonas aeruginosa. Urinary tract infection was diagnosed in 11 patients, corresponding to a hospital prevalence of 4%. Seven patients (63.6%) were female. No patient had signs of urinary urge. Of the 11 urinary tract infections diagnosed, E coli was isolated in eight (72.7%) cases. Klebsiella pneumoniae, Acinetobacter calcoaceticus and Enterobacter spp were found in one (9.09%) case each. Pneumonia was diagnosed in six patients, i.e. a prevalence of 2.22%. Men accounted for 83.3%. According to the British Thoracic Society prognostic score, all patients had a score greater than or equal to 2. In-hospital mortality was estimated at 83.3% in this population. Soft tissue infection such as leg erysipelas was diagnosed in seven patients, i.e. 2.5% of the study population. None of the patients had a fever on admission. Hepatic encephalopathy was present in all patients. Plasma albumin levels were below 25 g/L in 71.4% of cases. The Meld score was above 15 in all patients. Five patients (71.4%) died in hospital. A case of Klebsiella pneumoniae sepsis was diagnosed, corresponding to a prevalence of 0.37%. The patient was hospitalized for abdominal distension and fever. Physical examination on admission revealed stage 2 hepatic encephalopathy and grade 3 ascites. Renal function was impaired, with plasma creatinine at 24 mg/L. Of all the 14 germs isolated in our study, 4 (28.5%) were sensitive to Ceftriaxone, while the rest were sensitive to carbapenems. After univariate and multivariate analysis, a statistically significant association was found between the presence of ascites (*P*=0.017, OR=4.56), hepatic encephalopathy (*P*=0.032; OR=3.58), diffuse abdominal pain (*P*=0.017; OR=2.87), a prothrombin level below 25% (*P*=0.002; OR=9.67), a high Meld score (*P*=0.038; OR=0.93) and the occurrence of bacterial infection as shown in Table 3. There was no statistically significant difference between patients in-hospital survival with or without infectious complications, as shown in Figure 1.

**TABLE 1:** Comparative table of patients with and without bacterial infection.

	Infection n=63	No infection n=207	Total N=270	***P*-value**
Age in years (mean, SD)	50.4±12	49.2±13	49.5±12.9	0.5
Gender n (%)				
Male	45 (71.4)	134 (64.7)	179 (66.3)	0.3
Female	18 (28.5)	73 (35.2)	91 (33.7)	
Clinical characteristics n (%)				
Gastrointestinal bleeding	4 (6.35)	49 (23.6)	53 (19.6)	0.005
Abdominal pain	20 (31.7)	32 (15.4)	52 (19.2)	0.005
WHO status performance 3-4	42 (66.6)	101 (48.7)	143 (52.9)	0.014
Fever	14 (22.2)	25 (12)	39 (14.4)	0.05
Ascites	62 (98.4)	187 (90.3)	249 (77)	0.03
Hepatic encephalopathy	36 (57.1)	91 (43.9)	127 (47)	0.001
Biological characteristics				
Neutrophilic polynucleosis n (%)	32 (50.7)	69 (33.3)	101 (37.4)	0.024
Albumin levels g/L (mean, SD)	21.7±4.7	23.9±5.8	23.4±5.6	0.014
Intra-ascitic proteins <15 g/L n (%)	29 (50)	93 (62)	122 (45.1)	<0.001
Kidney failure n (%)	23 (36.5)	66 (31.8)	89 (32.9)	0.5
Prothrombin rate <25% n (%)	11 (17.4)	13 (6.2)	24 (8.8)	0.013
Etiology n (%)				
Chronic viral hepatitis	30 (47.6)	99 (47.8)	129 (47.7)	0.94
Alcohol consumption	27 (42.8)	91 (43.9)	118 (43.7)	
Prognosis				
Child-Pugh A n (%)	-	9 (4.4)	9 (3.4)	0.19
Child-Pugh B-C n (%)	63 (100)	198 (95.6)	261 (96.6)	
MELD score (median, IQR)	20 [16-29]	20 [14-26]	20 [14-48]	0.13
Length of stay in days (median, IQR)	10 [5-17]	8 [4-13]	8 [5-14]	0.01
Death n (%)	36 (57.1)	90 (43.4)	126 (46.6)	0.34

SD: standard deviation; IQR: interquartile range; MELD: Model for End stage Liver Disease.

**TABLE 2:** Prevalence of various infections in cirrhotic patients.

	n	%
Spontaneous bacterial peritonitis	**43**	15.9
Fever	12	27.9
Hepatic encephalopathy	19	44.1
Intra-ascitic proteins <15g/L	18	41.8
Child-Pugh C	30	69.5
Death	24	55.8
Urinary tract infection	**11**	4
Fever	3	27.2
Hepatic encephalopathy	6	54.5
Child-Pugh C	7	63.6
Escherichia coli	8	72.7
Death	4	36.3
Pneumonia	**6**	2.2
Fever	1	16.6
Hepatic encephalopathy	6	100
Child-Pugh C	6	100
Death	5	83.3
Soft tissue infection	**7**	2.5
Hepatic encephalopathy	7	100
Child-Pugh C	7	100
Death	5	71.4

**TABLE 3:** Factors associated with bacterial infection.

	Unadjusted results			Adjusted results		
	OR	***P*-value**	95%CI	OR	***P*-value**	95%CI
Ascites	5.79	0.2	0.44-185	4.56	0.017	1.47-18.6
Hepatic encephalopathy	4.32	0.024	1.22-16	3.58	0.032	1.10-11.7
Abdominal pain	3.05	0.018	1.20-7.8	2.87	0.017	1.20-6.85
Hyperleukocytosis	0.79	0.3	0.43-11.5	1.68	0.2	0.75-3.75
Prothrombin rate < 25%	12.1	0.002	2.59-65.3	9.67	0.002	2.36-43.9
Creatinine levels	1.37	0.13	0.91-2.12	1.40	0.065	0.99-2.04
Albumin	0.91	0.05	0.83-1	0.94	0.094	0.87-1;01
MELD score	0.90	0.2	0.76-1.07	0.93	0.038	0.86-O99

OR: odd ratio; 95%CI; 95% confidence interval.

## DISCUSSION

Bacterial infections in cirrhotic patients are common, favored by cirrhosis-induced immune dysfunction affecting both the innate and acquired immune systems^3^ ^,^ ^4^. However, there is little data on this subject in Sub-Saharan Africa. The overall prevalence of infections was 23.3%. This prevalence is underestimated because a complete infectious workup is not systematically performed in the event of acute decompensation, as recommended by the European Association for the Study of Liver (EASL)^5^. All cirrhotic patients with ascites on admission had their ascites examined systematically. As a result, all spontaneous bacterial peritonitis were community-acquired. The search for other sources of infection was not systematic in all patients on admission, mainly due to financial difficulties. Urine examinations and blood cultures were performed according to the patient’s clinical course. In that context, the distinction between community-acquired infection, healthcare-associated infection and nosocomial infection was not made, given the impossibility of carrying out the infectious workup within the required timeframe. Data on the prevalence of prophylactic antibiotic use prior to hospitalisation were not available because most patients pass through several health centers, where they receive treatment that is most often not specified. Patients with infection were all at the stage of decompensated cirrhosis, with 68% at stage C of the Child-Pugh prognostic classification. This result is identical to those reported in the literature and confirms that bacterial infections occur at the advanced stage of cirrhosis^3^ ^,^ ^6^. The germs isolated were all gram-negative bacteria, 64% of which were Escherichia coli, testifying to the enteric origin of the germs and confirming the role of bacterial translocation in the occurrence of bacterial infection^1^ ^,^ ^2^. In our study, the factors associated with bacterial infection were ascites (*P*=0.017, OR=4.56), hepatic encephalopathy (*P*=0.032; OR=3.58), diffuse abdominal pain (*P*=0.017; OR=2.87), a prothrombin level below 25% (*P*=0.002; OR=9.67) and a high Meld score (*P*=0.038; OR=0.93). This result is in line with the literature, which states that the risk factors for bacterial infections in cirrhotic patients are digestive haemorrhage, alcoholism and, above all, the degree of hepatocellular insufficiency^1^ ^,^ ^3^ ^,^ ^7^. As reported in the literature, spontaneous ascitic fluid infection was by far the most frequent infection during our study period, followed by urinary tract infection^8^ ^,^ ^9^. The predominance of spontaneous infection of ascites fluid is explained by the phenomenon of bacterial translocation, itself linked to increased intestinal permeability and lowered immune defences. Urinary tract infection was predominant in women (63.7%). This female predominance is similar to that of urinary tract infections in the general population. Child-pugh score and female gender were associated with urinary tract infection in several studies^10^ ^,^ ^11^. As in our study, Escherichia coli was the germ most frequently implicated in urinary tract infections in cirrhotic patients^1^. Cirrhotic patients are exposed to gram-negative bacillus and pneumococcal pneumonia, which justifies systematic vaccination in cases of cirrhosis^3^ ^,^ ^12^. Pneumopathy was classified as severe, requiring intensive care in all patients, with a high mortality rate (83.3%). A comparison of the prognosis of pneumopathy in patients with and without cirrhosis concluded that the severity and mortality were higher in the cirrhotic patient group^13^, which can be explained by the importance of immune dysfunction during cirrhosis. Soft tissue infection is favoured by the presence of lower limbs oedema, skin fragility, undernutrition and precarious hygiene condition^3^. The prevalence of soft tissue infection can be as high as 10% in some series^3^. In an Indian study, hypoalbuminemia below 25 g/L and a Meld score above 15 were associated with soft tissue infection^14^ ^,^ ^15^, as in our study. Bacterial infections are associated with a mortality rate 4 times higher than in the general population. However, in our study, there was no statistical difference between in-hospital survival of patients with or without infection. This can be explained by the fact that most patients in our study consulted at a late stage, which is responsible for a poor prognosis.

## CONCLUSION

Bacterial infections in cirrhosis are frequent, severe and highly fatal. They are linked to the degree of liver failure and are potentiated by the immune dysfunction induced by cirrhosis. Better knowledge of the microbial flora and the resistance profile of the responsible germs would help to better define the prophylaxis of these bacterial infections.
